# Thermal Rejection Assessment: New Strategies for Early Detection

**DOI:** 10.3389/ti.2025.14108

**Published:** 2025-04-16

**Authors:** Irina Filz von Reiterdank, Rohil Jain, Eloi de Clermont-Tonnerre, Alexandra Tchir, Curtis L. Cetrulo, Alexandre G. Lellouch, J. Henk Coert, Aebele B. Mink van der Molen, Shannon N. Tessier, Korkut Uygun

**Affiliations:** ^1^ Department of Surgery, Center for Engineering for Medicine and Surgery, Massachusetts General Hospital, Harvard Medical School, Boston, MA, United States; ^2^ Shriners Children’s Boston, Boston, MA, United States; ^3^ Department of Plastic, Reconstructive and Hand Surgery, University Medical Center Utrecht, Utrecht University, Utrecht, Netherlands; ^4^ Vascularized Composite Allotransplantation Laboratory, Massachusetts General Hospital, Harvard Medical School, Boston, MA, United States; ^5^ Department of Plastic, Reconstructive and Aesthetic Surgery, Hôpital Paris Saint-Joseph, Paris, France

**Keywords:** rejection, transplantation, infrared, FLIR, vascularized composite allografts

## Abstract

Skin pigmentation can pose challenges for physicians to diagnose pathologies. In Vascularized Composite Allotransplantation (VCA), this increases the difficulty of diagnosing rejection by clinical observation, which could be improved by noninvasive monitoring, thereby completely avoiding or aiding in guiding location for invasive diagnostics. In this study, pigmented and non-pigmented allogeneic and non-pigmented syngeneic control transplant recipients underwent daily thermal assessment using infrared (IR) gun and forward-looking IR (FLIR) imaging of VCAs using a rodent partial hindlimb transplant model. Daily clinical assessment was performed, and biopsies were taken on postoperative day (POD) 1, 3, and 7. Clinical and histological assessments indicated signs of rejection on POD 3. In contrast, thermal assessment using the IR gun detected significant differences as early as POD 1, notably a decrease in temperature, when comp ared to syngeneic control transplants. This demonstrates the capability of thermal assessments to identify early signs of rejection before clinical symptoms become apparent. The findings suggest that thermal assessments can serve as a non-contact, objective adjunct tool for early detection of graft rejection, with consideration of skin pigmentation. This approach may reduce the need for invasive biopsies, thereby improving patient comfort and reducing potential complications associated with current diagnostic methods.

## Graphical Abstract

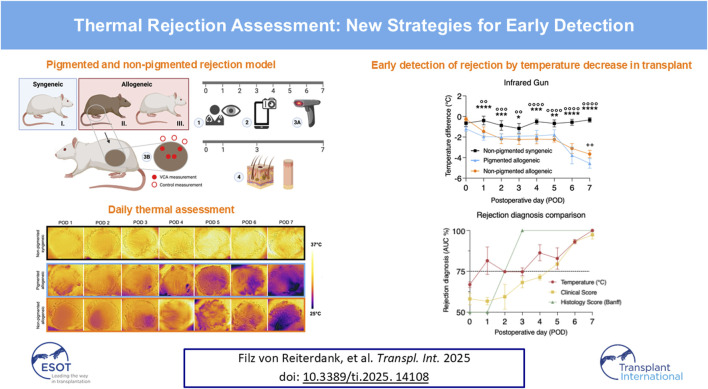

## Introduction

Early diagnosis of acute rejection is essential for the immunological management of transplant patients, affecting comorbidity, chronic rejection, and risk of complete graft failure [1]. Transplants involving skin are especially high-risk due to the immunogenic nature of skin tissue, and acute rejection episodes occur in 89% of patients [2]. Traditionally, diagnosis relies on serial biopsies and clinical observation [3]. Biopsies are risky and painful, while visual assessment of the skin can be imprecise and subjective, especially in pigmented skin where early signs of rejection, such as erythema, are less apparent [4–6]. Early detection of changes in graft health can lead to prompt treatment, reducing the severity of rejection episodes and potentially avoiding complete graft failure [1]. By developing additional non-invasive and objective methods, VCA’s surface-level accessibility can be leveraged for more effective early detection and monitoring.

This study introduces an innovative engineering solution that uses thermal imaging to non-invasively and diagnose acute rejection in a rat model of VCA transplantation in a few seconds using affordable commercial devices. Infrared (IR) gun for point measurements, and forward-looking IR (FLIR) imaging technologies are used to offer a reliable adjunct tool to use across two distinct skin pigmentation levels. Both technologies record the graft surface temperature by analyzing the emitted IR from the graft in the 8 to 14 microns wavelength range. Predicated on the thermodynamic principles of heat transfer from the blood circulation to the graft, these measurements may serve as an indirect measure of skin perfusion and, consequently, graft viability with correlation to early stages of graft rejection. Non-invasive imaging has been suggested in the past to determine rejection and avoid serial biopsies, often involving blood flow assessment, visual markers after intravenous injection, or stiffness measurements using ultrasound and MRI techniques [7]. In comparison, the IR approach is fast, portable, quantitative, and particularly valuable in resource-limited settings due to its straightforward application and cost-effectiveness, thereby addressing a critical gap in skin diagnostics and reconstructive transplant surgery. However, the majority of existing studies have not studied skin pigmentation as a variable, thus potentially limiting the applicability of the technology and excluding the needs of all affected patients.

Skin-containing transplantations, which play a crucial role in reconstructive surgery, exemplify the challenges at the intersection of skin pathology and transplant medicine. Vascularized Composite Allotransplantations (VCAs), auto-transplantations, free flap transfers, and sentinel skin flaps, while innovative, are often hindered by the difficulty in early detection of complications when using subjective clinical observations, especially in pigmented skin [8]. Far from being a challenge unique to VCAs [3], such disparities are representative of a broader issue in the field of transplantation and medical diagnostics in general. Amongst others, race and ethnicity greatly determine the chance of referral for transplant evaluation, being added to the waiting list, and receiving a transplant [9]. Recent attempts to address challenges with pigmented patients have sometimes included adding more invasive procedures, placing a greater burden on the patient. For example, the first Black patient to receive a face transplant underwent additional mucosal biopsies, which were not typically required for other patients [8]. Considering these observations, inadequate diagnostic tools and sluggish technological development contribute to discriminatory practices [10] and non-invasive alternatives may be found to prevent unnecessary procedures in all patients.

By focusing on thermal parameters, this study aims to develop a method that is effective in transplant surgery. In doing so, this study investigates temperature assessment as an effective, non-invasive early detection tool for graft rejection using a rodent VCA transplantation model, suitable across different skin types. The aim is to facilitate early, accessible, and straightforward intervention irrespective of skin pigmentation, leading to improved clinical outcomes and more equitable healthcare.

## Materials and Methods

### Animals

60 rats (male, 250 ± 50 g) were used for all experiments, of which 42 were inbred Lewis rats, 11 Brown Norway rats, and 7 Buffalo rats (Charles River Laboratories, Wilmington, MA). The animals received humane care in accordance with the National Research Council guidelines and the experimental protocols were approved by the IACUC of Massachusetts General Hospital (Boston, MA).

### Study Design

**FIGURE 1:**
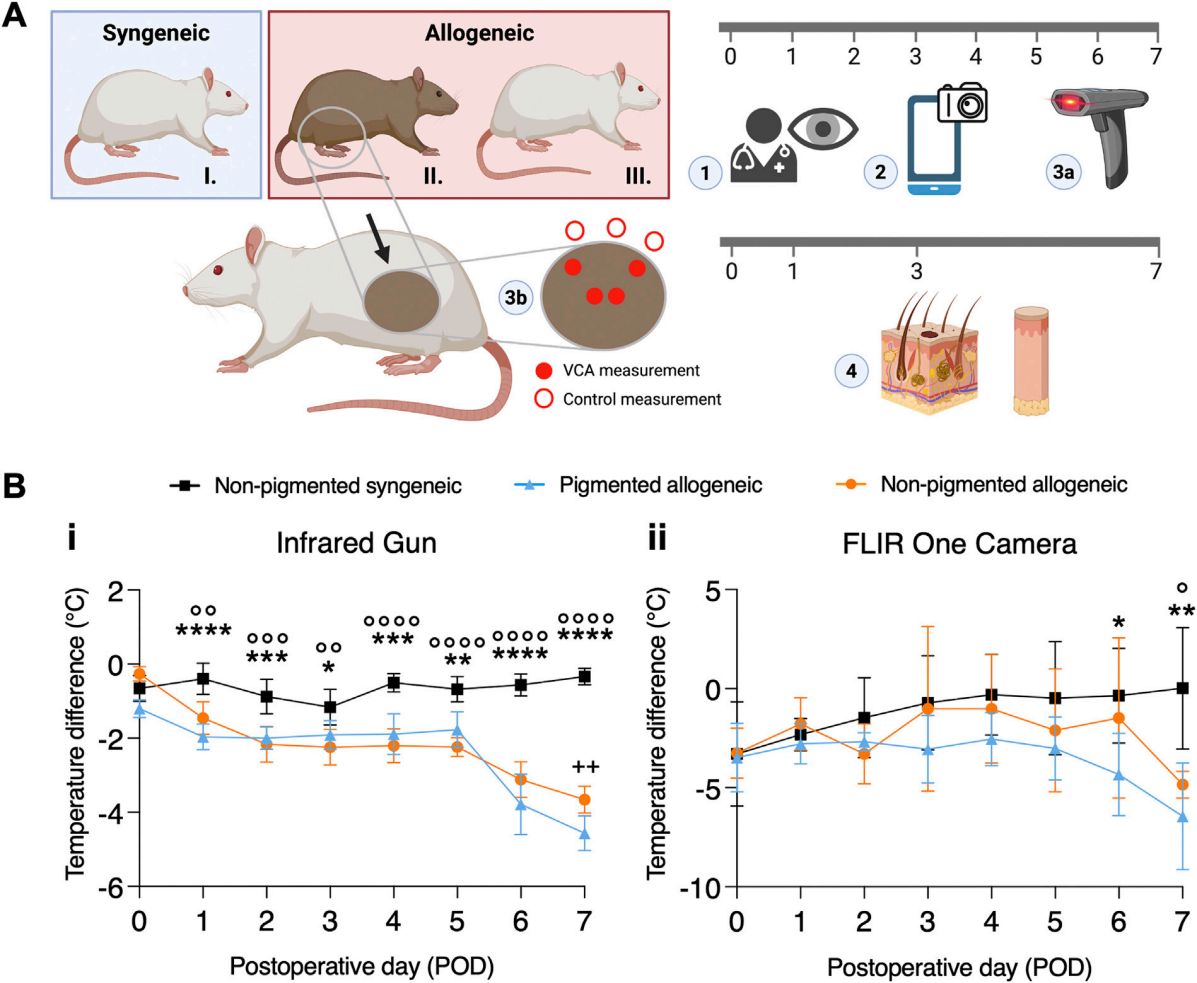
Experimental design and temperature difference between transplant and native skin over time. **(A)** Partial hindlimb transplant model for the following three groups: I. non-pigmented syngeneic, II. pigmented allogeneic, and III. non-pigmented allogeneic transplants. Blue and red boxes at the top represent the donors in each group, while the non-pigmented animal at the bottom represents the recipients used for all groups. On the timeline, the modality and frequency of assessments are indicated. Daily (1) clinical assessment was performed by experienced surgeons, (2) Smartphone-based FLIR One images, and (3a) gun-style infra-red (IR) thermometer measurements were taken. (3b) IR gun measurements were performed daily, while (4) histology was obtained on postoperative day (POD) 0, 1, 3, and 7 biopsies were taken and assessed by a blinded pathologist. IR gun measurements were obtained from the center and periphery of the graft for adequate sampling, in addition to control measurements outside of the graft. **(B)** Temporal variation in temperature difference between VCA and native skin are displayed as mean and error ((95% CI). (i) Shows variation in temperature difference measured using IR gun, with a statistically significant difference between pigmented allogeneic (n = 8) and non-pigmented syngeneic control (n = 12, denoted using *), as well as between the non-pigmented allogeneic (n = 7) and syngeneic control groups (n = 12, denoted using °). Apart from POD7, no significant difference is found between the pigmented and non-pigemented allogeneic groups (denoted using ^+^) (ii) Temperature assessment using FLIR shows a similar trend despite the lack of sensitivity. */° p ≤ 0.0332; **/°°/^++^ p ≤ 0.0021; ***/°°° p ≤ 0.0002; ****/°°°° p ≤ 0.0001.

Partial hindlimb transplants were performed in three different surgical groups (Figure 1A) [1]: pigmented allogeneic (rejection) group (n = 11) in which Brown Norway rats were donors [2]; non-pigmented allogeneic (rejection) group (n = 7) in which Buffalo rats were donors [3]; non-pigmented syngeneic control (no rejection) group (n = 12) in which Lewis rats were donors. In all transplants Lewis rats were recipients. Buffalo and Lewis rats are considered albino animals therefore would be considered Fitzpatrick skin type I. Brown Norway rats have a non-Agouti brown coat meaning they are solid-colored. To our knowledge, no equivalent scale to the Fitzpatrick skin types exists for rats, however, we would consider Brown Norway rats to be closest to a Fitzpatrick skin type IV-V. Use of pigmented animal models with similar immunological compatibility allows for cross-pigmentation measurements on the same timeline providing positive and negative control groups.

### VCA Transplantation

After induction using isoflurane (5%) inhalation with 100% O_2_, general anesthesia was sustained with inhaled isoflurane (1%–3%) and anesthesia depth was confirmed with a toe pinch test. Partial hindlimbs were procured as described earlier [11]. Briefly, grafts include the knee joint with 10 mm distal femur and 10 mm proximal femur and tibia, along with thigh muscle groups with the inguinal fat pad and calf muscles as well as the surrounding skin paddle. Femoral vessels were skeletonized and ligated 5 min after IV administration of 100 IU/mL/kg heparin in the penile dorsal vein. The femoral artery was cannulated with a 24G angio catheter and secured with a 6/0 nylon suture. The femoral vein was cut after ligation. Immediately after procurement, a pressure-controlled manual flush with 3 mL (200IU) of heparin saline at room temperature was performed. Next, the VCA was transplanted into a Lewis rat. Recipient vessels were prepared on the contralateral side in a similar fashion to the donor. Vessels were ligated distally and prepared for anastomosis. A longitudinal incision in the flank was made with subsequent tunneling to the groin area for VCA insertion. Femoral arteries and veins were anastomosed using a self-developed adjusted cuffing technique to allow for application to partial hindlimb transplant. Skin on the donor VCA was excised to create an oval flap in the flank which was secured with interrupted 5-0 sutures. Inguinal fat pad and groin skin incision were similarly closed with interrupted 5-0 vicryl sutures.

### Postoperative Assessments

Postoperatively, daily flap images were taken for blinded clinical assessment by six blinded clinicians using a clinical VCA rejection score. Briefly, grade 0 constitutes no difference between graft and native skin. Grade 1 shows mild erythema, grade 2 moderate erythema with beginning of scaling and scabbing, grade 3 severe erythema and scabbing with areas of epidermolysis, and grade 4 constituting full-thickness graft epidermolysis with areas of necrosis. Temperature measurements were taken daily as displayed in Figure 1A using a temperature IR gun (Digisense, Cat. N° 20250-07) and FLIR thermal images (FLIR ONE^®^ Pro – iOS). Both devices were held at approximately 20 cm distance to the region of interest. Gun measurements were taken of the center and periphery of the flap, control measurements were taken of the skin immediately dorsal to the flap. FLIR images were taken of the entire flank area. For analysis, the mean temperature of the flap area and the mean of an area immediately dorsal of the flap was taken in a blinded fashion. Diurnal variations in body temperature were accounted for by control measurements of surrounding native skin in the same animal, ensuring the reliability of the results by reducing environmental influences on the temperature.

### Histology

On postoperative day (POD) 1, 3, and 7 skin and muscle biopsies were taken (Figure 1). On POD 7 additional muscle biopsies were taken. Biopsies were fixed in formalin and processed for histopathological examination. Slides were stained with hematoxylin and eosin (H&E). A blinded evaluation by a pathologist was performed for all biopsy samples and using the Banff criteria score to assess acute cell-mediated rejection [12, 13]. Briefly, grade 0 is considered no rejection, grade I mild (mild perivascular infiltration, no involvement of epidermis), grade II moderate (moderate perivascular infiltration, possible mild epidermal involvement), grade III severe (dense inflammation and epidermal involvement) and grade IV necrotizing acute rejection (frank necrosis of the epidermis and other skin structures). For the skin samples, a mean Banff score was calculated for comparison. Muscle tissues were evaluated and scored using the histology injury scoring system (HISS) for hypoxia-induced muscle injury [14].

### Statistical Analysis

Temperature data is analyzed using a linear mixed effects model with the type of transplant (3 levels; pigmented allogeneic, non-pigmented allogeneic, non-pigmented syngeneic) and POD (8 levels; POD 0-7) as fixed effects while also accounting for their interaction. Locations on the flap (3-4 per subject) and subjects (7–12 per condition) were treated as random variables for the temperature gun data. For FLIR data, average temperature for the whole flap is used for analysis, thereby only subject is treated as the random variable. Multiple comparisons were performed using Tukey’s corrected multiple comparisons test with 8 families (one for each time point). The appropriateness of the model was confirmed with a residual plot that showed no correlation of the residuals with the predicted values, and the normality assumption was confirmed with a QQ plot that showed high coincidence between the predicted and actual residual values (Supplementary Figure S1).

Discriminative performance of thermal assessment for detecting graft rejection in the early PODs (POD 1 and 2) was evaluated using two separate methods. Firstly, a linear mixed effects model with type of transplant (3 levels as described above) and only early PODs (2 levels; POD 1, and POD 2) as the fixed effects are used while accounting for their interaction. For the discriminatory analysis, post-hoc analysis using multiple comparisons with Tukey’s correction is performed under the assumption of one family for the entire transplant type. Secondly, a binary classification system is applied, and corresponding receiver operating characteristic (ROC) curves are generated, that independently compare two pairings: pigmented rejection with the non-rejection group, and the non-pigmented rejection group with the non-rejection group. The binary classifiers also utilize temperature values from POD 1 and 2 for each pairing type. Furthermore, the effectiveness of each pairing is compared for each individual POD.

Clinical rejection score differences between groups were analyzed using a mixed-effects model with multiple comparisons. The time-series plots are represented as mean with 95 Confidence Interval (CI), bar charts are represented as mean with Standard Deviations (SD). All statistical analyses were performed using Prism 9 for Mac OSX (GraphPad Software, La Jolla, CA). p-Values less than 0.0332 were considered to be significant.

## Results

Transplants in all three groups were successful until end of study as defined by visual assessment using the vascular patency test.

### Postoperative Thermal Trend Analysis Indicates Rejection Can Be Detected as Early as Day 1

Representations of daily clinical images are shown in Supplementary Figure S2A and corresponding FLIR images are shown in Supplementary Figure S2B, which readily reveal visual indicators of graft rejection in a pigment-agnostic manner as early as POD1. Temperature difference between VCA and surrounding native skin using the IR gun (Figures 1B-i) was assessed using a mixed effects model as described in the methods section and found significant effect of both the fixed effects and their interaction (p < 0.0001). The standard deviation for the random effects (subject x location) is 0.51. The model was also found to have highly effective matching, indicating that the mixed effects model was the appropriate choice for analysis (p < 0.0001). Furthermore, post-hoc analysis to compare means for each POD shows a significant difference between the pigmented (p < 0.0001) and non-pigmented (p = 0.0068) rejection groups compared to the non-rejection group from POD 1 onwards. While the level of significance fluctuates and shows a decrease on POD3 in both groups, it remains significant until the end of study. FLIR temperature assessment (Figures 1B-ii) shows a similar trend in mixed effects analysis (fixed effects and interaction significant with p < 0.05, matching effective at p < 0.0001, SD of random effect: 1.17) as well as post-hoc multiple comparisons, even though statistically significant differences are not observed until POD 6.

### Infrared Gun Shows Superior Sensitivity and Specificity Compared to FLIR

**FIGURE 2:**
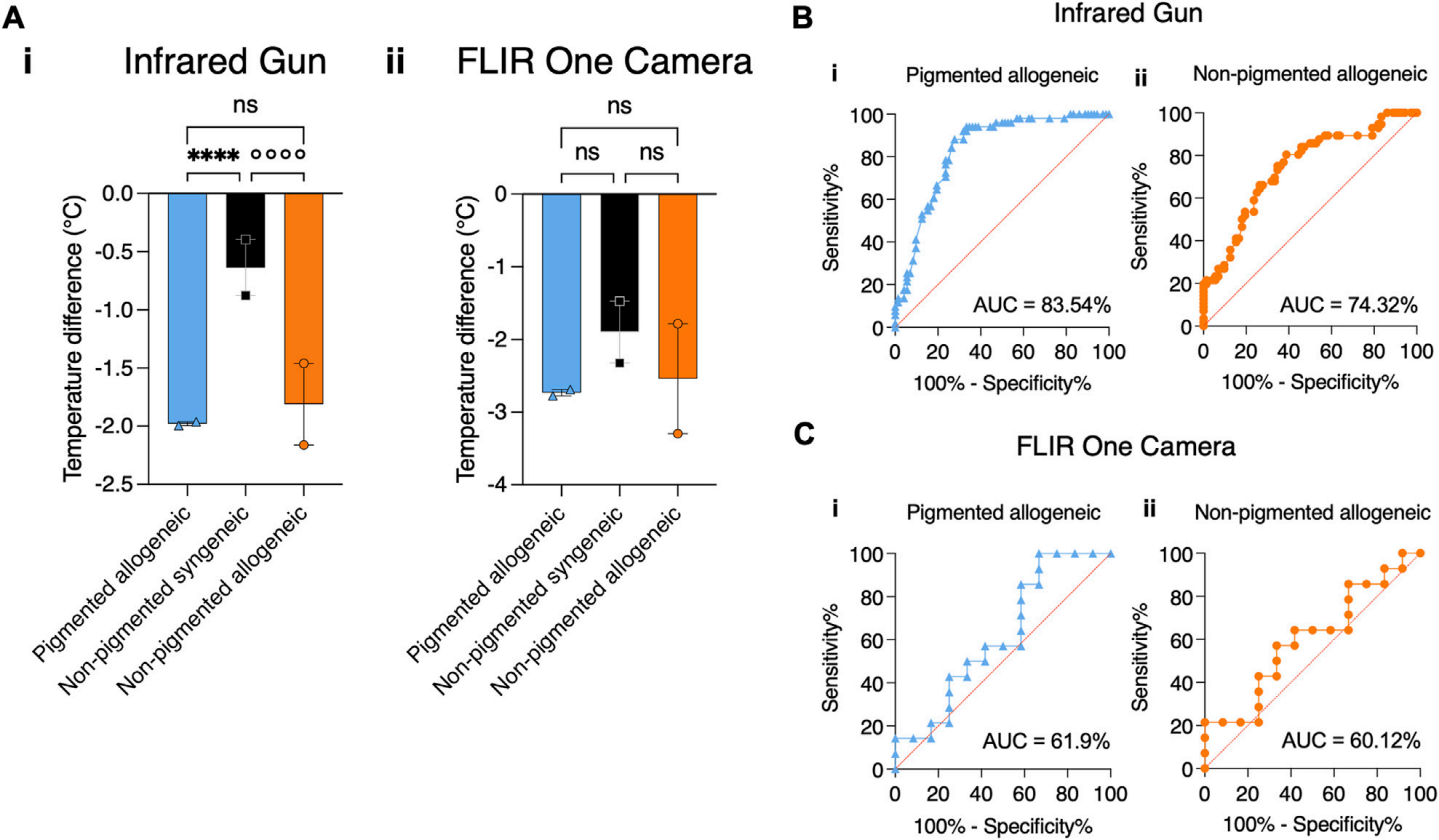
Comparing the effectiveness of Infrared gun versus FLIR One Camera to detect temperature differences in transplanted graft using statistical tests. **(A)** Average temperature differences between VCA and native skin are shown for all groups by combining measurements from POD1 and 2. (i) Temperature difference measured by IR gun shows statistical significance between allogeneic and syngeneic groups regardless of pigmentation. (ii) Temperature measured by FLIR One Camera does not show statistical significance between allogeneic and syngeneic groups for all pigmentation levels, despite showing a similar trend as IR gun. **(B)** For the same time points, a binary classifier analysis shows a high Area Under the Curve (AUC) for a receiver operating characteristic (ROC) curve using the IR gun in detecting rejection in both the (i) pigmented (83.54%) and (ii) non-pigmented (74.32%) groups. **(C)** A similar binary classifier analysis using the FLIR One Camera shows a lower AUC of (i) 61.9% in the pigmented and of (ii) 60.12% in the non-pigmented rejection group. ****/°°°° p ≤ 0.0001.

Figure 2A shows that thermal assessment indicated significant differences between rejection and non-rejection groups as early as POD 1 and 2, however only in the case of IR gun the average temperature difference reached statistical significance. Fitting of the mixed effects model on the data from the IR gun showed a statistically significant effect of the fixed effects, i.e., POD and type of transplant (p < 0.005), however, no effect of interaction between POD and transplant type was found (p > 0.05), allowing for grouping POD 1 and 2 data for post-hoc comparison. Tukey’s corrected multiple comparison for temperatures showed statistically significant difference between each of the rejection groups with the non-rejection group (p < 0.0001). Neither the mixed effects model, nor the post-hoc comparison for the data from the FLIR measurements reached statistical significance (p > 0.05). Correspondingly, AUC analysis reflects a higher sensitivity and specificity of IR gun measurements than FLIR measurements with an AUC of 83.54% in the pigmented group and 74.32% in the non-pigmented group using combined IR gun temperature data from POD 1-2 (Figures 2B, C). AUC analysis of all other PODs is shown in Supplementary Figure S3, S4. Similar to the daily thermal trend analysis, daily AUC curves show some fluctuation. To minimize data dependence on daily fluctuations in Figure 2A temporal component was integrated by using the average of POD 1 and 2.

### Clinical Assessment Does Not Diagnose Rejection Before Day 3

**FIGURE 3:**
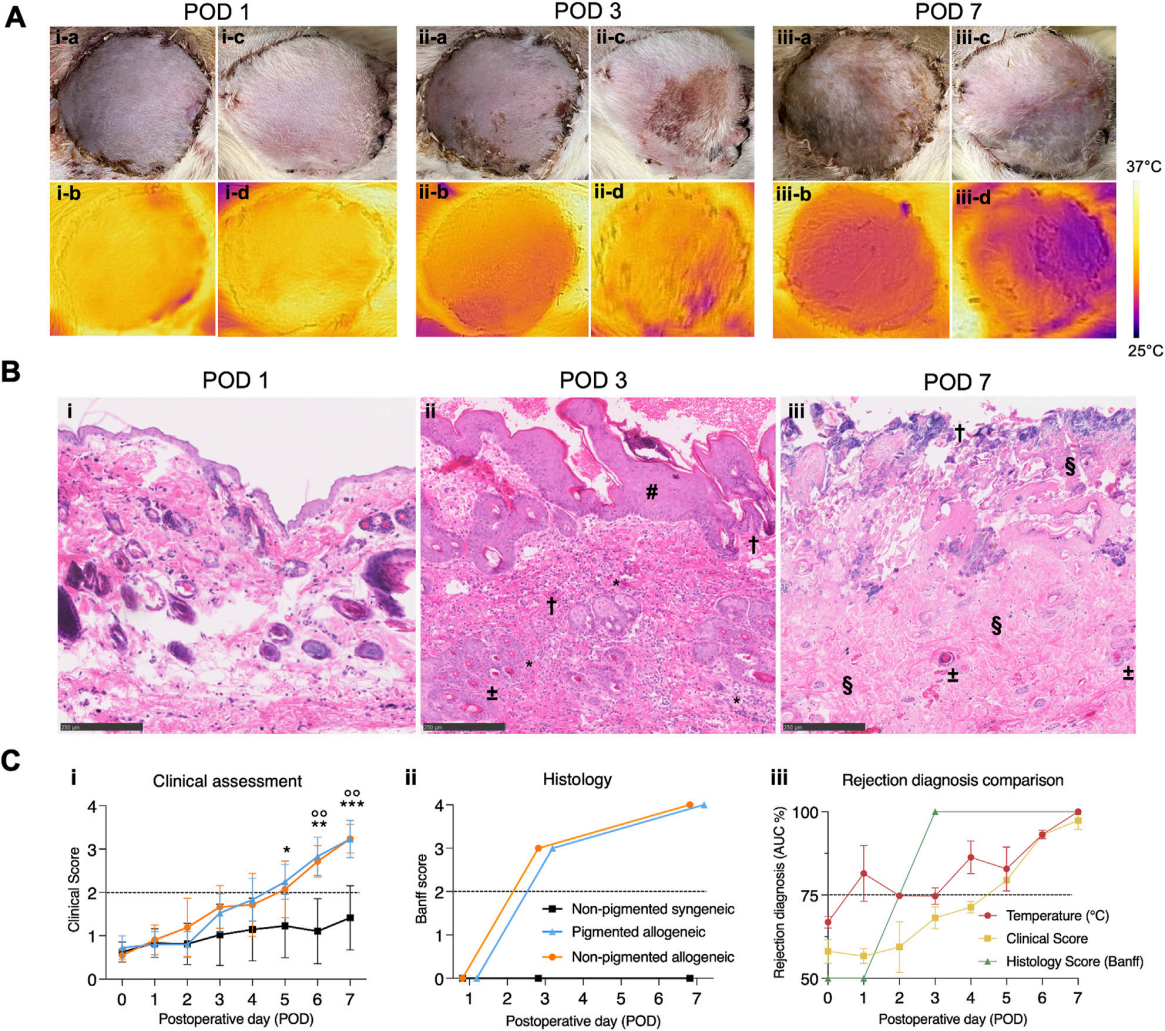
Comparison of time to diagnosis between visual, thermal, and histological assessment methods for pigmented and non-pigmented allogeneic groups. **(A)** (i) Representative images in visible spectrum for clinical evaluation and IR spectrum show indistinguishable differences with both methods on POD1. (ii) A drop in temperature of the VCA compared to native skin is seen on POD3 in both groups. Simultaneous clinical evaluation shows subtle, erythema and epidermolysis which is clearly distinguishable in the non-pigmented group. (iii) By POD7, rejection is pronounced in both pigmented and non-pigmented allogeneic groups, as observed clinically by features of epidermolysis, necrosis, and lymphatic fluid oozing. Temperature difference of the VCA is also more pronounced in both pigmented and non-pigemented allogeneic groups. **(B)** Microscopic analysis of histology with H&E staining and bright field microscopy (scale bar 250 µm) shows (i) no abnormal features on POD1. (ii) At POD3, focal epidermal necrosis is observed in both rejection groups with epidermal thickening (#), infiltration (*), microthrombi (±) and apoptotic bodies (†). (iii) By POD7, full-thickness skin necrosis (†) with severe loss of architecture (§) and thrombi (±) is seen in the rejection groups. **(C)** Analysis of clinical assessment scores and histology grading shows (i) rejection is identifiable at a slightly earlier time in the non-pigmented group (ii) Conversely, blinded microscopic Banff evaluation shows no significant differences between groups and shows more severe rejection than clinical assessment suggests. Above the dotted black line indicates moderate to severe rejection. (iii) Association between temperature assessment, clinical rejection score, and histological Banff score (daily ROC curve based AUC of individual data points for each type of assessment versus POD curve) shows that temperature assessment has an earlier association with rejection than both other scores (dotted line at 75%). * p ≤ 0.0332; **/°° p ≤ 0.0021; *** p ≤ 0.0002.

Representations of daily clinical images are shown in Figure 3A, and corresponding histological images in Figure 3B. In both rejection groups at POD 1, the mean clinical assessment score was 0.25 (±0.21), indicating minimal observable changes at this early stage. In the non-pigmented group the mean score was 0.35 (±0.03), while the pigmented group was only scored at a mean of 0.15 (±0.33). At POD 3, the mean score increased to 1.65 (±0.14), suggesting grafts show mild to moderate erythema with some showing the beginning of scaling and scabbing. Similarly, the non-pigmented group was scored lower at 1.22 (±0.28). By POD 7, the mean score of both rejection groups further increased to 3.47 (±0.04), reflecting pronounced clinical signs of severe erythema with areas of epidermolysis and necrosis or crust, consistent with graft rejection. This far into the rejection process, mean scores between the rejection groups were more similar with 3.57 (±0.27) in the non-pigmented group and 3.2 in the pigmented group. The mean day on which rejection was clinically diagnosed was at 2.71 ± 0.44) and 2.96 (±0.35) in the non-pigmented and pigmented grafts respectively, highlighting slightly earlier diagnosis in the non-pigmented group compared to the pigmented group. In the non-rejection group, grafts showed normal postoperative recovery signs which could be confused with early stages of rejection, however, none of the grafts showed high clinical rejection scores, as expected.

### Histological Assessment Does Not Detect Rejection Before Day 3

Histology at POD 1, 3, and 7 is shown in Figure 3C-ii and its analysis in Figure 3C-iii. In both experimental groups, on POD 1, no pathological findings related to rejection were detected in skin tissue. Muscle tissue showed mild to moderate ischemic changes as displayed in Supplementary Figure S5. At POD 3, skin samples showed focal epidermal necrosis resulting in a Banff score of III in both experimental groups. Muscle tissue showed moderate edema and inflammation. By POD 7, a Banff score of IV was found in both experimental groups based on severe ischemic changes with early necrosis of muscle tissue and full-thickness skin necrosis, indicative of advanced histological rejection, as shown in more detail in Supplementary Figure S6. In the non-rejection group, no pathological signs were found in skin nor muscle tissue. No significant differences were found between the non-pigmented and pigmented rejection groups.

### Comparison Between Thermal, Clinical, and Histological Assessment

As shown in Figures 3C-iii, for the experimental groups combined it was observed that the daily AUC for temperature assessment (72.22–91.22) was consistently higher than the AUC for clinical scoring (54.46–73.08) from POD 1 until POD 4. This aligns well with our hypothesis that temperature-based assessment can provide an early measurement of the comorbidities associated with rejection. Further, the AUC of the rejection groups for temperature assessment and clinical scores are high and align well for POD 5 to POD 7 (76.29–100 and 78.57 to 100, respectively), with both techniques predicting rejection with very high confidence and accuracy.

## Discussion

This study presents a comprehensive examination of the utility and sensitivity of thermal assessment techniques (IR gun and FLIR imaging) in the early detection of acute rejection in a rodent VCA model in a pigmentation-agnostic manner. The presented findings may indicate a potential role of thermal assessment is more effective in early detection than clinical assessments, which often fail to detect rejection in pigmented skin until POD 3. In contrast, thermal assessment shows significant differences between rejection and non-rejection groups as early as POD 1, irrespective of skin pigmentation.

### Technical Requirements of IR Technology

The use of IR technology for temperature measurement, while straightforward, has surprisingly not played a larger role in clinical practice, nor have temperature profiles of transplant organs been extensively studied. One reason for this may be that it is only in recent years that this technology has achieved affordability, accuracy, and compactness for medical use. Both IR gun and FLIR camera offer significant advantages in the <$500 price range, where the gun provides higher accuracy for point measurements, whereas the FLIR camera allows spatial coverage of the graft at some loss in accuracy. Additionally, FLIR cameras also require significant post-processing to obtain an average temperature of the whole graft. In this study, comparative analysis between similarly priced IR gun ($350) and FLIR imaging camera ($400) revealed that both IR gun and FLIR imaging follow a similar trend of changes in temperature for POD 0–7. However, the FLIR image-based analysis does not reach significance in early graft rejection analysis. It is likely because the resolution of the FLIR One camera (±3°C) that was used was insufficient to capture the small-scale differences between allogeneic and syngeneic grafts. For instance, the multiple comparisons test showed a mean temperature difference of at least 0.27°C between the allogeneic groups compared to the syngeneic group on all the PODs. The necessary precision of temperature measurement is likely pathology-dependent [15, 16]. The FLIR One smartphone thermography has been used successfully in clinic [17], however, some applications, such as presented in this study and others [18], will require higher precision.

### Research to Practice: Sensitivity and Specificity

When we started the study, our original hypothesis was, that a temperature increase would be found in the VCAs in the early stages of rejection, followed by a temperature decrease in the later stages of rejection. This hypothesis was based on the knowledge that endothelial activation during acute rejection can lead to vasodilation (e.g., bradykinin, prostacyclin, nitric oxide), while the activated complement system and pre-formed DSAs can trigger intravascular coagulation [19, 20]. However, our study demonstrated that rejection leads to a significant temperature decrease in VCAs as early as POD 1. The decrease in VCA temperature during rejection found may be a result of impaired microcapillary perfusion and, therefore, disrupted heat distribution. This observation is similar to the only other study that examined temperature changes during rejection in a kidney transplantation model using an implantable bioelectric device [21]. Here, continuous temperature monitoring showed a temperature increase, followed by a sharp decrease in temperature, which worsened until graft loss. It is possible that due to the full mismatch model used in our study, and the use of daily measurements rather than continuous measurements, an early rise in temperature within the first 24 h was not recorded. Mechanistic studies are required to differentiate between confounding pathologies for a drop in graft temperature, similar temperature profiles for rejection across disparate organ systems (kidney and VCA) point to the potential utility of thermal assessment of organ transplantation in general. A large animal model may be more appropriate for such work, which would also allow sequential tissue biopsies for time series analyses; this is not feasible in a small animal model since the graft size does not lend itself for multiple biopsies. For VCAs this is especially relevant in the acute phase during which high rejection rates remain a challenge [2, 22].

In our controlled laboratory setting, thermal assessment demonstrated high sensitivity and specificity compared to current subjective diagnostic methods [23]. However, additional confounding factors such as patient-to-patient variability, environmental conditions, and surgical complications may need to be accounted for in a clinical trial with patients.

### Emerging Applications

Thermal assessment of skin has been suggested as a diagnostic and monitoring tool for various conditions characterized by altered skin perfusion, such as assessing burn wounds [24, 25], evaluating vessel patency in peripheral arterial disease [26], monitoring surgical flap viability [27–30], and detecting perfusion anomalies associated with tumor growth [31]. Cherchi et al. [32] even proposed a potential intra-operative role for thermography for the detection of signs of early graft dysfunction. Furthermore, the use of sentinel skin transplants has been suggested as a rejection detection tool in solid organ transplantation [33, 34], with recent reports of first clinical case results [35]. The non-contact nature of the technique is highly suitable for immediate clinical translation, as a supportive approach to enhance prediction of rejection. For future studies, we recommend to assessing long-term follow-up and evaluating the effects of immunotherapy and its withdrawal in larger animal models or by immediately incorporating this diagnostic into a clinical trial. A potential clinical plan would involve several key steps: first, measure temperature profiles in autologous skin, VCA, and free flap transfer transplants to establish standard temperature benchmarks for all patients, ideally involving a cohort of different pigmentation levels. This would effectively be a control group for non-rejection graft monitoring. The next phase would involve testing temperature profiles in allogeneic VCA patients and sentinel flap clinical trials (currently ongoing) [36] to further validate its effectiveness. Moreover, for application to research, thermal assessment has been mentioned as a technique to increase standardization and reproducibility in burn wound models and the effect of treatment in these models [37].

### Limitations

Several limitations, such as moderate sample size, possible differences in skin architecture, and immunological behavior between rat and human VCA tissues remain [38]. Acute rejection has a heterogeneous distribution, as FLIR images reveal temperature variations and injury in specific areas of the flap. Despite the limitations of a small-size rodent model, the proportionate graft area is significant relative to the total body size. Refining FLIR techniques could better guide biopsies than IR gun measurements. In the FLIR images of rejection at POD 3–5, we are able to see hotspots of temperature variations within the flap. Variations across the flap could become more pronounced in larger animals or bigger flaps, which accentuates the complex and potentially localized nature of rejection, offering opportunities for more precise and targeted interventions. Depending on the application and chosen various approaches for thermal assessments can be usedm such as monitoring absolute temperature [21], identifying hot spots [39], or comparing the temperature of region of interest with surrounding native skin [25, 40].

### Conclusion

This study demonstrates that a temperature decrease is found in rejecting grafts in rodents, which can be detected early, non-invasively, and objectively, independent of the presence of skin pigmentation. The results suggest that there may be a role for thermal assessment in improving patient outcomes and postoperative care as well attempting to contribute to a reduction of health disparities. For clinical trials involving thermal imaging, future studies should address the lack of skin color variation in rodent animal models by considering the wide range of pigment differences across various racial and ethnic groups to ensure representative and inclusive recruitment. Additionally, translation to other skin pathologies can provide a more general diagnostic tool for pigmented skin. This way, physicians can be guided in the clinical decision-making process and minimize invasive, costly, and time-consuming diagnostic tools for patients.

An early detection capability is critical in the context of transplant surgery, where early intervention can significantly impact patient outcomes. Assessment techniques independent of skin pigmentation, such as shown in this study, offer a more inclusive approach to clinical care. To our knowledge, this study represents the first thermal analysis of allogeneic VCAs including analysis of pigment-dependence. It is shown that significant differences in graft temperature are found as early as POD 1 and 2, while clinical and histological assessment is delayed until POD 3, especially in pigmented grafts. Furthermore, a minimum sensitivity is needed to detect significant changes. The detection is low-cost and does not require extensive training. The results show promise for thermal assessment as an objective, quantifiable, non-invasive, easy-to-use, and quick adjunct tool for early rejection detection in a pigment-agnostic manner.

## Data Availability

The raw data supporting the conclusions of this article will be made available by the authors, without undue reservation.

## Abbreviations

AUC, Area Under the Curve; FLIR, Forward-Looking Infra-Red; H&E, Hematoxylin and Eosin; IR, Infra-Red; POD, Post Operative Day; ROC, Receiver Operating Characteristic; VCA, Vascularized Composite Allografts.
