# Supplementary material for: Thermal Rejection Assessment: New Strategies for Early Detection
Source: Transpl Int. 2025 Apr 16;38:14108. doi: 10.3389/ti.2025.14108 (PMC12040617; doi:10.3389/ti.2025.14108)
Supplement: Supplementary file 1 [file DataSheet1.docx]

**Supplemental materials**

**
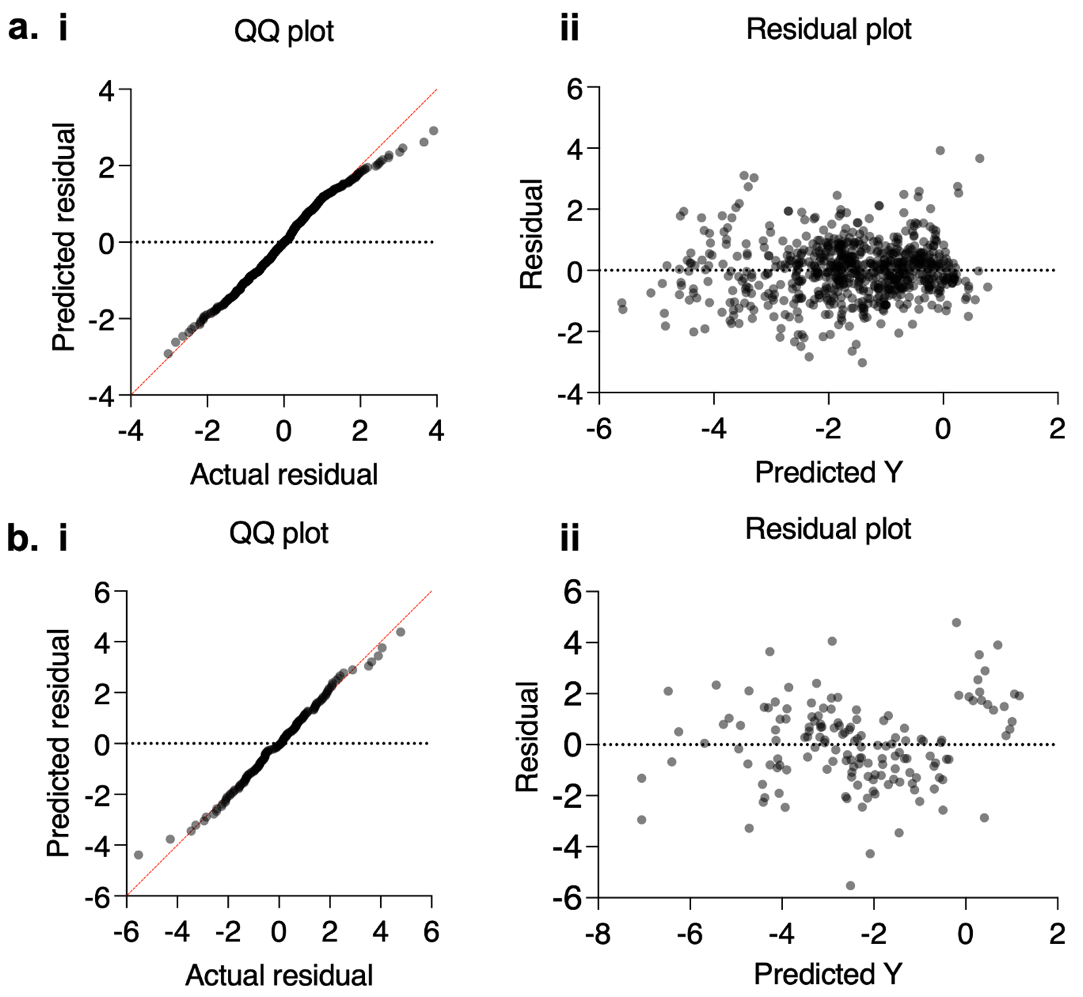
**

**Figure S1. QQ-plot and Normality Assumption.** For both (**A**) IR gun and (**B**) FLIR images, the appropriateness of the model was confirmed with a (**A/B-i**) residual plot that showed no correlation of the residuals with the predicted values, and (**A/B-ii**) the normality assumption was confirmed with a QQ-plot that showed high coincidence between the predicted and actual residual values.


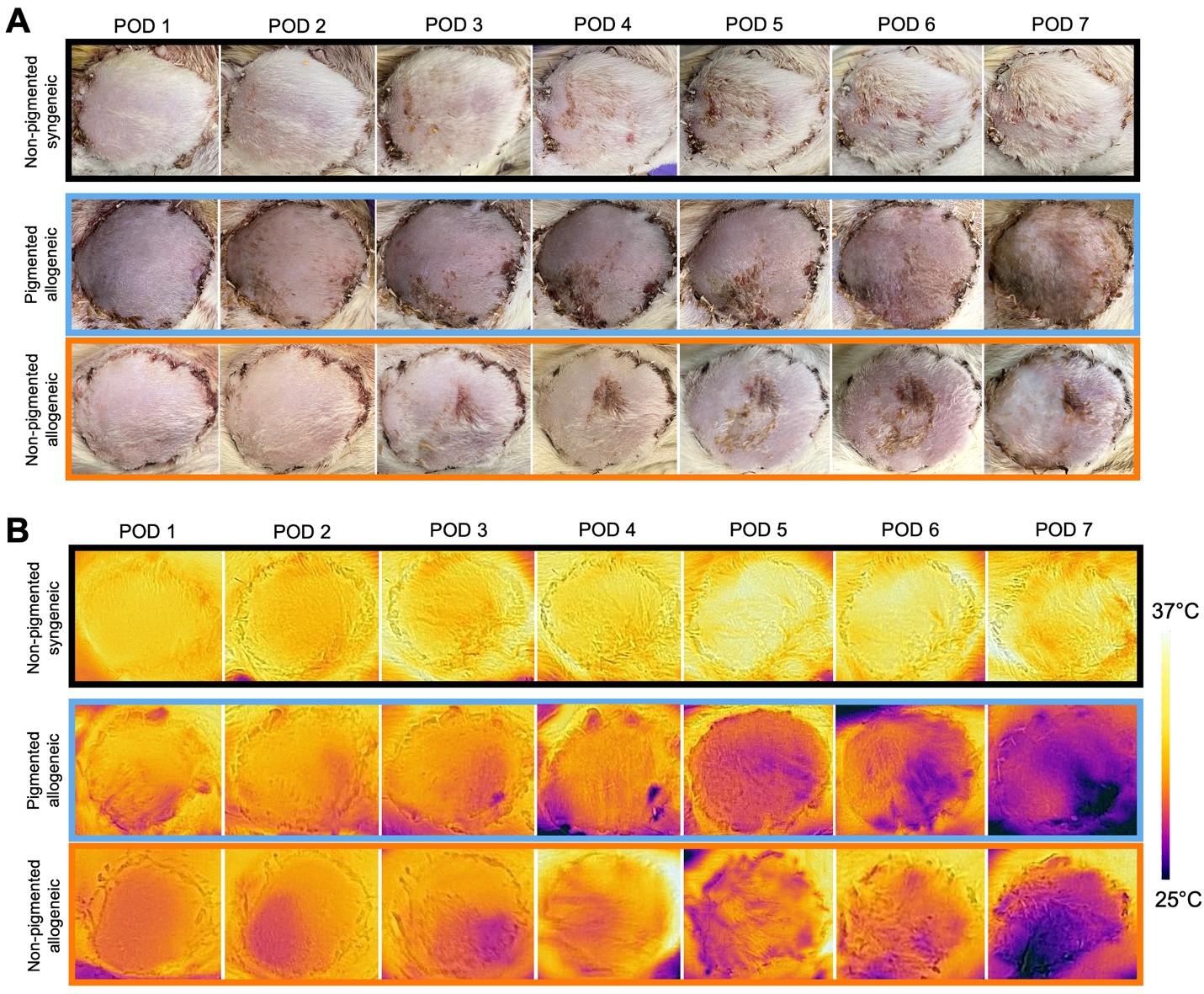


**Figure S2. Representative daily images of the VCAs.** (**A**) Daily clinical assessment for each group shows VCA development in non-rejection and rejection models. (**B**) Similarly, representative daily FLIR images for temperature assessment for each group are shown.

**
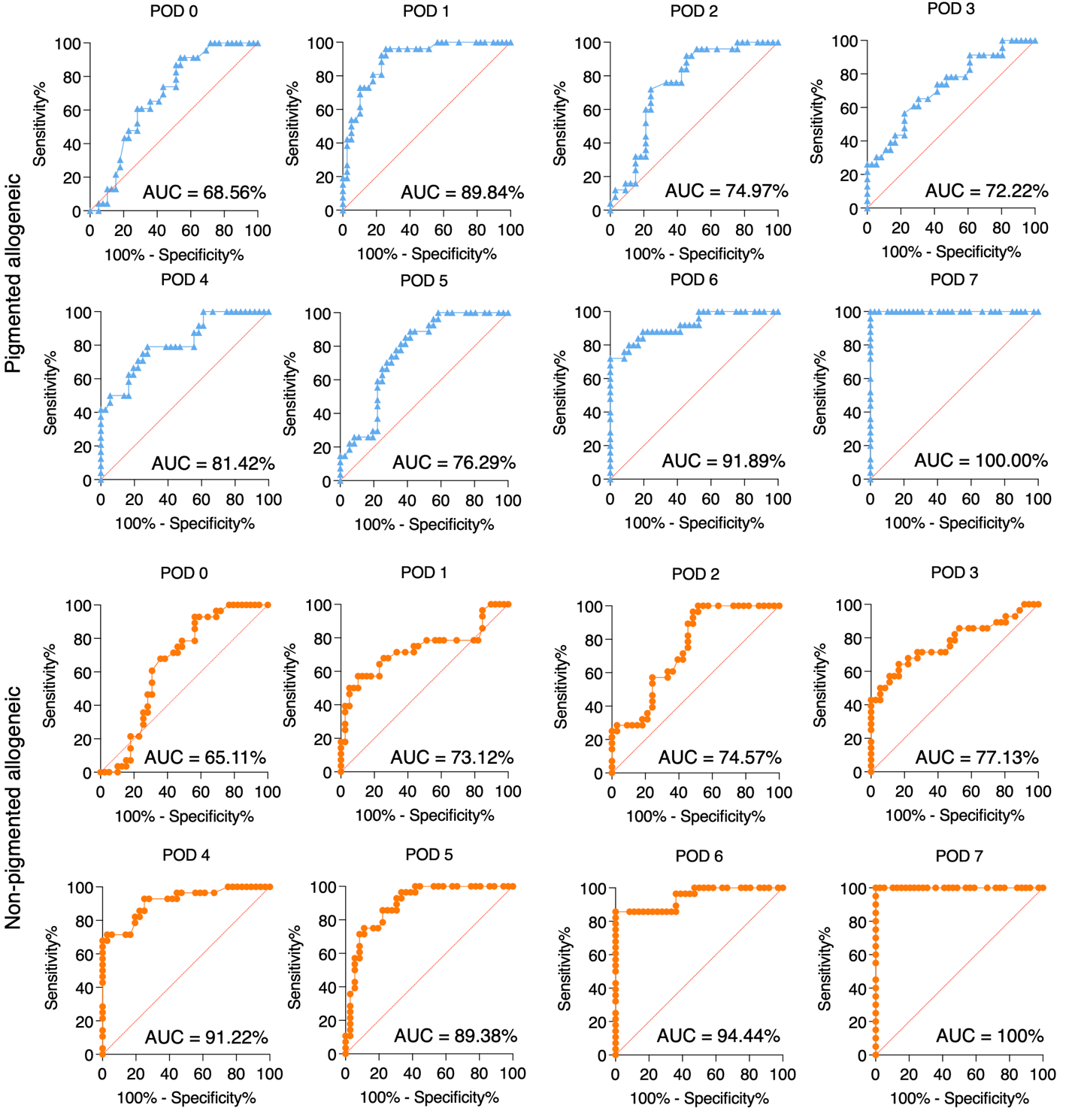
**

**Figure S3. Daily AUC curves of IR gun measurements.** Daily average temperature differences measured by IR gun show significant differences between rejection and non-rejection groups regardless of pigmentation, which increases as the rejection worsens. For the same time points, AUC analysis shows high specificity/sensitivity of temperature using the IR Gun in detecting rejection in both the pigmented and non-pigmented group from POD 1 onwards.

**
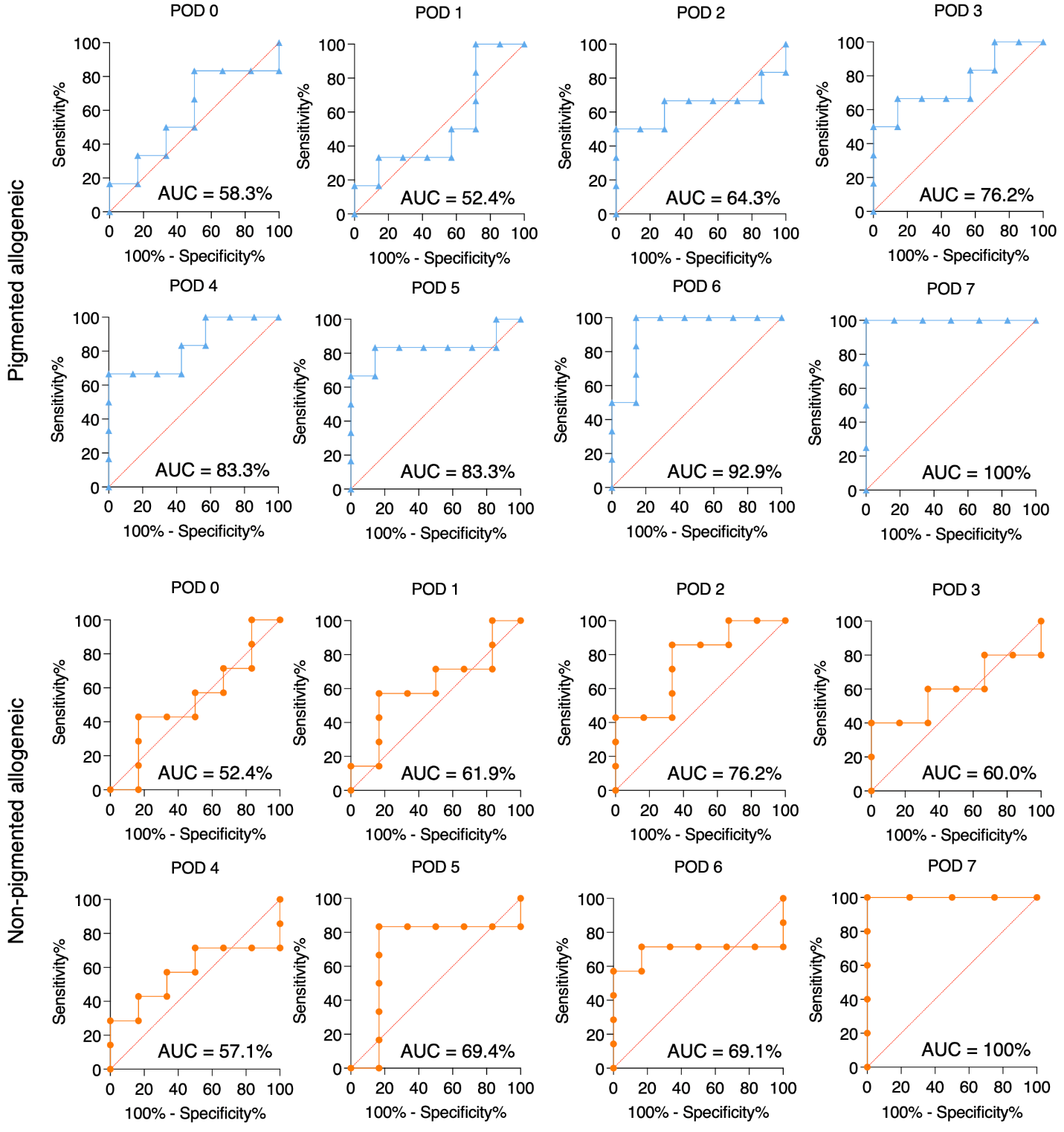
**

**Figure S4. Daily AUC curves of FLIR camera measurements.** Daily AUC curves of thermal assessment using FLIR show a similar trend as the IR gun. However, significance is not reached until POD 6.


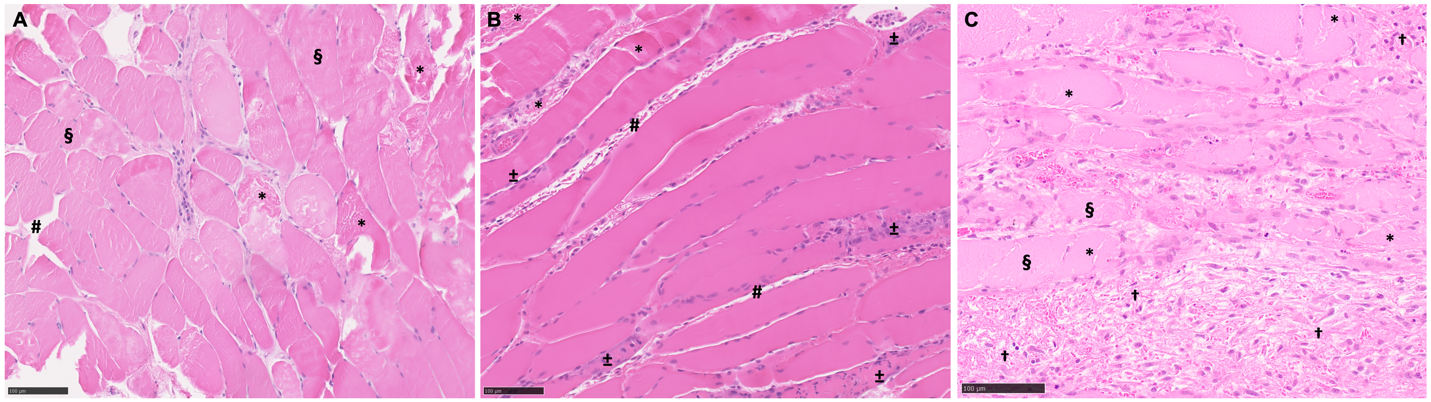


**Figure S5. Histological evaluation of graft muscle biopsies.** Longitudinal and cross-section, H&E staining (scale bar 100 µm). (**A**) At POD 1, muscle samples in the rejection groups show mild to moderate signs of ischemia with myocyte size variation (**§**) and myocyte damage (*****). (**B**) At POD 3, signs of rejection are seen with moderate edema (**#**), myocyte damage (*****), and inflammation (**±**). (**C**) By POD 7, severe ischemic changes are seen with early necrosis (**†**).


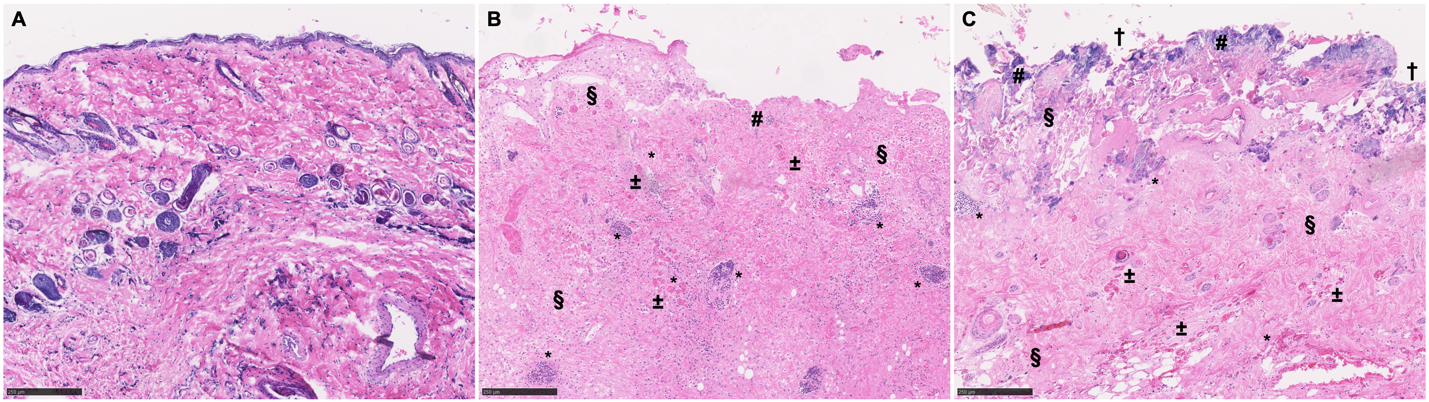


**Figure S6. Histological evaluation of all transplanted group on day 7.** Longitudinal and cross-section, H&E staining (scale bar 250 µm). (**A**) Syngeneic transplants (n = 12) show no signs of rejection, normal architecture, and presence of skin adnexa on POD 7 (Banff 0). (**B**) In contrast, both pigmented allogeneic (n = 9) and (**C**) non-pigmented allogeneic (n = 11) show immune cell infiltration (*), apoptotic bodies (#), microthrombi (±), full-thickness skin necrosis (†) with severe loss of architecture (§) (Banff IV) in all replicates on POD 7.
